# Epidemiology and risk factors of chronic kidney disease in India – results from the SEEK (Screening and Early Evaluation of Kidney Disease) study

**DOI:** 10.1186/1471-2369-14-114

**Published:** 2013-05-28

**Authors:** Ajay K Singh, Youssef MK Farag, Bharati V Mittal, Kuyilan Karai Subramanian, Sai Ram Keithi Reddy, Vidya N Acharya, Alan F Almeida, Anil Channakeshavamurthy, H Sudarshan Ballal, Gaccione P, Rajan Issacs, Sanjiv Jasuja, Ashok L Kirpalani, Vijay Kher, Gopesh K Modi, Georgy Nainan, Jai Prakash, Devinder Singh Rana, Rajanna Sreedhara, Dilip Kumar Sinha, Shah Bharat V, Sham Sunder, Raj K Sharma, Sridevi Seetharam, Tatapudi Ravi Raju, Mohan M Rajapurkar

**Affiliations:** 1 Brigham & Women’s Hospital & Harvard Medical School, 75 Francis Street, Boston, MA 02115, USA; 2 NKF, Mumbai, India; 3 Hinduja Hospital, Mumbai, India; 4 Vivekananda Memorial Hospital, H.D.Kote, Saragur, Mysore, India; 5 Manipal Institute of Nephrology & Urology, Bangalore, India; 6 Deep Hospital, Ludhiana, India; 7 Indraprastha Apollo Hospital, New Delhi, India; 8 Bombay Hospital, Mumbai, India; 9 Fortis Flt. Lt. Rajan Dhall Hospital, New Delhi, India; 10 Bhopal Memorial Hospital & Research Center, Bhopal, India; 11 PVS Memorial Hospital, Cochin, India; 12 Institute of Medical Sciences, BHU, Varanasi, India; 13 Muljibhai Patel Urological Hospital, Nadiad, India; 14 Sir Ganga Ram Hospital, New Delhi, India; 15 Wockhardt Hospitals, Bangalore, India; 16 Kanpur Rotary Kidney Foundation, Kanpur, India; 17 Lilavati Hospital, Mumbai, India; 18 Dr. R.M.Lohia Hospital, New Delhi, India; 19 Sanjay Gandhi Postgraduate Institute of Medical Sciences, Lucknow, India; 20 Andhra Medical College & King George Hospital, Vishakhapatanam, India

**Keywords:** Chronic kidney disease, Prevalence, Epidemiology, Risk factors, SEEK, Screening programs, India, Diabetes, Hypertension, South East Asia

## Abstract

**Background:**

There is a rising incidence of chronic kidney disease that is likely to pose major problems for both healthcare and the economy in future years. In India, it has been recently estimated that the age-adjusted incidence rate of ESRD to be 229 per million population (pmp), and >100,000 new patients enter renal replacement programs annually.

**Methods:**

We cross-sectionally screened 6120 Indian subjects from 13 academic and private medical centers all over India. We obtained personal and medical history data through a specifically designed questionnaire. Blood and urine samples were collected.

**Results:**

The total cohort included in this analysis is 5588 subjects. The mean ± SD age of all participants was 45.22 ± 15.2 years (range 18–98 years) and 55.1% of them were males and 44.9% were females. The overall prevalence of CKD in the SEEK-India cohort was 17.2% with a mean eGFR of 84.27 ± 76.46 versus 116.94 ± 44.65 mL/min/1.73 m2 in non-CKD group while 79.5% in the CKD group had proteinuria. Prevalence of CKD stages 1, 2, 3, 4 and 5 was 7%, 4.3%, 4.3%, 0.8% and 0.8%, respectively.

**Conclusion:**

The prevalence of CKD was observed to be 17.2% with ~6% have CKD stage 3 or worse. CKD risk factors were similar to those reported in earlier studies.

It should be stressed to all primary care physicians taking care of hypertensive and diabetic patients to screen for early kidney damage. Early intervention may retard the progression of kidney disease. Planning for the preventive health policies and allocation of more resources for the treatment of CKD/ESRD patients are imperative in India.

## Background

Chronic kidney disease (CKD) is emerging to be an important chronic disease globally [1]. One reason is the rapidly increasing worldwide incidence of diabetes [2] and hypertension [3,4]. In India, given its population >1 billion, the rising incidence of CKD is likely to pose major problems for both healthcare and the economy in future years. Indeed, it has been recently estimated that the age-adjusted incidence rate of ESRD in India to be 229 per million population (pmp) [5], and >100,000 new patients enter renal replacement programs annually in India [6]. On the other hand, because of scarce resources, only 10% of the Indian ESRD patients receive any renal replacement therapy (RRT) [6-8]. The lack of community-based screening programs has led to patients being detected with CKD at an advanced stage. It is possible that early detection of kidney disease through community based screening programs might have an impact on this problem through earlier intervention. The Screening and Early Evaluation of Kidney Disease Project (SEEK) was designed and performed to generate data to determine the prevalence and risk factors for CKD in India.

## Methods

Thirteen academic and private medical centers in India participated in the study under the name of “Screening and Early Evaluation of Kidney disease - SEEK.” Any Indian male and female with age over 18 years are eligible to participate in the screening. It was conducted between June 2005 to May 2007, coordinated from the Brigham and Women’s Hospital in Boston, Massachusetts, a teaching affiliate of Harvard Medical School. The protocol was approved by the Partner’s Institutional Review Board (IRB) as well as by individual centers own institutional IRBs. Signed or verbal informed consent (confirmed by a witness) was obtained before administering the questionnaire, taking measurements or blood collection. The database is based at the Brigham and Women’s Hospital.

### Questionnaire

A structured questionnaire was developed and pre-tested in the pilot study of 500 subjects carried out at one center in South India (Additional file 1). The questionnaire was translated into local languages (e.g., Telugu, Hindi, Marathi, Kannada, Gujarati and Malayalam). The questionnaire was generally administered by non-medical staff /volunteers who were trained by the SEEK-India team in interviewing techniques. At every site, staff was trained in interview techniques and measurement of height, weight and blood pressure by organizing a half day workshop prior to the camp. A team of nephrologists, nurses, technicians and trained interviewers participated in the camps. Questionnaires collected from the field were reviewed by the local site principal investigator and data entry was carried out locally.

### Anthropometric measures

Body mass index (BMI) was calculated using the formula “weight (Kg) / height (m2).” The waist to hip circumference ratio (WHR) was calculated by using the waist circumference at the narrowest circumference between the lower costal margin and the iliac crest. Hip circumference was measured at the maximum circumference at the level of the femoral trochanters.

### Blood pressure measurement

In order to get a standardized blood pressure (BP) measurement, a protocol per American Heart Association guidelines [9] and a power-point presentation was provided to the centers, and staff training was carried out prior to camps. Systolic blood pressure (SBP) was based on the 1st Korotkoff phase and diastolic (DBP) on the 5th Korotkoff phase. Mercury sphygmomanometer was used after checking for zero error. BP was recorded in the sitting position in the right arm supported at heart level, to the nearest 2 mm using mercury sphygmomanometer. An average of two readings was taken into consideration.

### Blood and Urine sample collection

Random blood samples were collected. Blood was sent to a central laboratory. Quality control for temperature transporting specimens was checked and confirmed; i.e., 4–9 degree Celsius. Serum creatinine was measured using Jaffe Colorimetric method on a Roche Hitachi 912 analyzer. The instrument was calibrated (external calibration) using the Cleveland Clinic Foundation (CCF) creatinine panel. Regression analysis was carried out to calculate a formula to convert creatinine values obtained at the SRL-Ranbaxy laboratory (SRL) to the CCF values as follows: CCF creatinine = −0.13 + SRL creatinine * 0.99. Urine protein was detected by dipstick method (Bayer Multistix 10 SG). A modified MDRD-3 equation GFR(mL/min/1.73 m2) = 175 × (Scr)^- 1.54^ × (Age)^- 0.203^ × (0.742 if female) × (1.212 if African American) was used [10]. Plasma glucose was measured by the glucose oxidase peroxidase method using Roche Hitachi 912 analyzer.

### Variables definitions

Hypertension was defined as SBP/DBP > = 140 /90 mm of Hg [11] or if the patient was on medication for hypertension or had a positive self reported history of hypertension (based on a response to “have you ever been told that you have high blood pressure” or “a past history of high blood pressure”).

Diabetes was defined as fasting blood sugar FBS > = 126 or random blood sugar > = 200 or on any medications for diabetes mellitus (ADA definition) [12], or if there was a positive response to the questions “have you ever been told that you have diabetes” or “past history of diabetes.” Self reported history of medications was verified and if the subject did not know the name of the medication and or if the stated name was incorrect, the response was considered as “no” even if the subject’s response to the question on “are you on BP or diabetes medications” was “yes.”

According to the International Diabetes Federation (IDF) criteria, abdominal obesity was defined waist circumference of ≥94 cm in men and ≥80 cm in women [13].

CKD stages were defined using NKF-KDOQI guidelines (eGFR < 60 ml/min/1.73 m2 or proteinuria > 1+ on dipstick) [14]. Urine protein positivity (proteinuria) was defined as urine albumin 1+ or more. We used the CKD-EPI equation [15] in the sensitivity analysis to further explore the burden of CKD using this equation.

Self reported ischemic heart disease was taken as present if there was a self reported history of a myocardial infarction, percutaneous angioplasty or coronary artery bypass surgery (CABG).

All patients with CKD diagnosed in camps received the reports along with a referral to the local hospitals/clinics.

### Statistical analysis

IBM SPSS Statistics for Windows, version 19, 2010, SPSS Inc., an IBM company, was used. In the descriptive analysis, continuous variables were expressed as mean ± standard deviation (SD) and categorical variables were expressed as count (percentages). While making no assumption about the distribution of data, normality distribution testing of the continuous variables was performed using the non-parametric test; Kolmogorov-Smirnov test (KS-test). Univariate analyses comparing distributions of socio-demographic and clinical/historical measures between CKD groups was performed using independent unpaired student t Test for normally distributed continuous variables and Mann–Whitney U Test for non-normally distributed continuous variables. For categorical variables we used Pearson chi-square test. Fisher’s exact test was used when there was one or more of cells with an expected frequency <5. We performed spearman correlation analysis to study the relationship between eGFR and other variables. The values of Spearman’s rho and the p-value have been reported.

## Results

**Figure 1:**
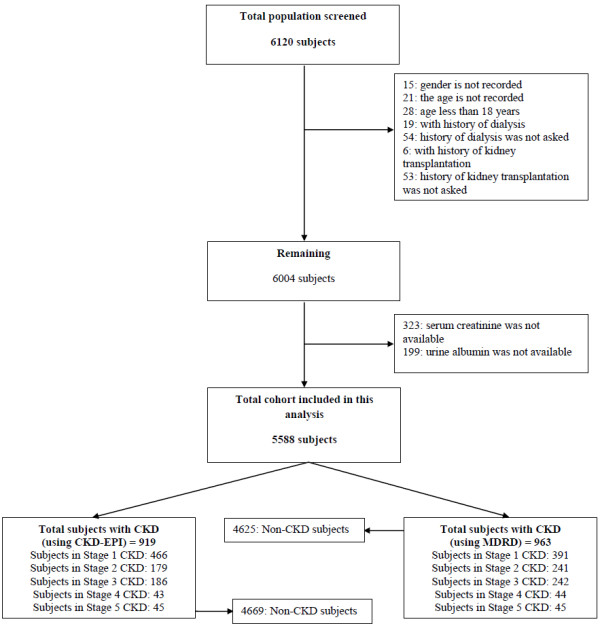
Flowchart of the SEEK-India Cohort.

Six thousand one hundred and twenty subjects were screened as part of the SEEK-India project. We recruited subjects through 53 screening camps in 12 cities across India representing almost all Indian regions (Table 1). We excluded subjects who were less than 18 years of age (n = 28), with history of dialysis (n = 19) and with history of kidney transplantation (n = 6) (Figure 1). We further excluded subjects for which certain variables’ results were not recorded. These variables included gender, age, history of dialysis or kidney transplantation, serum creatinine and urine albumin. The total cohort included in this analysis is 5588 subjects. The mean ± SD age of all participants was 45.22 ± 15.2 years (range 18–98 years) and 55.1% of them were males and 44.9% were females. Hypertension was observed in 43.1% of our population while 18.8% of them were diabetic. The mean ± SD of BMI was 23.91 ± 5.3 kg/m2. Defining overweight and obesity as BMI between 25–30 and >30 kg/m2, respectively, the prevalence of overweight and obesity in our sample was 26.4% and 11.7%, respectively. However, 36.5% had abdominal obesity where the mean waist circumference was 83.03 ± 14 cm. The mean estimated glomerular filtration rate (eGFR) using the MDRD-3 and CKD-EPI equations were 111.31 ± 53 and 104.9 ± 25.52 mL/min/1.73 m^2^ respectively. The remaining baseline demographics, clinical and laboratory data were summarized in Tables 2 and 3.

**Table 1:** Distribution of subjects among screening centers and regions

**Region and center**	**Number of subjects recruited**
**North India**	**2298 (36.7)**
Varanasi, Uttar Pradesh	515 (8.8%)
Kanpur, Uttar Pradesh	511 (9.1%)
Delhi	752 (10.9%)
Himachal Pradesh	519 (7.9%)
**Northwest Inida**	**402 (6.9%)**
Ludhiana, Punjab	402 (6.9%)
**Central India**	**438 (7.4%)**
Bhopal, Madhya Pradesh	438 (7.4%)
**Western India**	**1037 (17%)**
Nadiad, Gujarat	506 (8.9%)
Bombay, Maharashtra	531 (8.1%)
**Southwest India**	**1794 (29.5%)**
Mysore, Karnataka	1022 (17.1%)
Bangalore, Karnataka	275 (4.5%)
Cochin, Kerala	497 (7.9%)
**Southeast India**	**152 (2.5%)**
Visakhapatnam, Andhra Pradesh	152 (2.5%)
**Total**	**6120**

**Table 2:** Baseline demographic and risk factors data for SEEK India Cohort

	**All participants**	**By CKD status (using MDRD)**			**By CKD status (using CKD-EPI)**		
		**CKD**	**Non-CKD**	***p***	**CKD**	**Non-CKD**	***p***
**Age (in years) (n = 5588)**	45.22 ± 15.2	52.27 ± 14.78	43.75 ± 14.88	<0.0001	52.19 ± 14.9	43.85 ± 14.89	<0.0001
**Gender**							
**Male**	55.1%	61%	53.9%	<0.0001	62.5	53.7	<0.0001
**Female**	44.9%	39%	46.1%		37.5	46.3	
**Education**							
**< High school**	43.6%	37.6%	45.1%	<0.0001	37.1	45.2	<0.0001
**> = High school**	56.2%	62.4%	54.9%		62.9	54.8	
**Residence status (urban)**	49.7%	73.4%	44.7%	<0.0001			
**Income (<$125/month)**	63.7%	52.6%	69%	<0.0001			
**Present or Past Smoker**	19.9%	21.2%	20%	0.227	21.5	20	0.158
**Overweight (BMI 25–30 kg/m**^**2**^**)**	26.4%	31.6%	25.5%	<0.0001	31.6%	25.5%	<0.0001
**Obesity (BMI > = 30 kg/m**^**2**^**)**	11.7%	16.8%	10.7%	<0.0001	16.6	10.8	<0.0001
**Abdominal obesity**	36.5%	48.1%	36.4%	<0.0001	47.8%	36.6%	<0.0001
**Diabetes**	18.8%	31.6%	16.1%	0.000	32.2	16.1	<0.0001
**HTN**	43.1%	64.5%	41.9%	<0.0001	64.6	42.1	<0.0001
**Anemia**	33.1%	40.7%	31.8%	<0.0001	41.1	31.8	<0.0001
**History of Ischemic heart disease**	5.2	6.9	4.8	0.007	7	4.8	0.006
**History of stroke**	1.3%	2.6%	1%	<0.0001	2.5	1	0.001
**History of Hypercholesterolemia**	5.3%	7.7	4.8	<0.0001	7.6	4.8	0.001
**History of PVD**	2.4%	3	2.9	0.847	3	2.9	0.973
**History of TB**	4.6%	3.1	4.9	0.015	3.3	4.9	0.033
**History of kidney stones**	4.5	5.3	4.3	0.196	5.2	4.4	0.254

**Diabetes**: FBS > = 126 mg/dL or RBS > =200 mg/dL or self-reported or positive medication for diabetes confirmed by a physician.

**HTN**: BP > = 140/90 mmHg or self-reported or positive medication for hypertension confirmed by a physician.

**Anemia**: NKF definition: hemoglobin level <13.5 g/dl in men and 12.0 g/dl in women.

**IHD**: self-reported history of IHD or angioplasty or CABG.

**PVD**: Peripheral Vascular Disease.

**Table 3:** Clinical and laboratory data for the SEEK India cohort

	**All participants**	**By CKD status (using MDRD)**			**By CKD status (using CKD-EPI)**		
		**CKD**	**Non-CKD**	***p***	**CKD**	**Non-CKD**	***p***
**Weight (kg)**	60.74 ± 14.3	63.15 ± 14.68	60.24 ± 14.2	<0.0001	63.26 ± 14.75	60.24 ± 14.19	<0.0001
**Height (cm)**	159.4 ± 10.3	158.7 ± 10.2	159.52 ± 10.3	0.062	158.87 ± 10.9	159.47 ± 10.32	0.276
**BMI (kg/m**^**2**^**)**	23.91 ± 5.3	25.12 ± 5.67	23.66 ± 5.2	<0.0001	25.1 ± 5.69	23.68 ± 5.2	<0.0001
**Waist circumference (cm)**	83.03 ± 14	87.01 ± 14.4	82.26 ± 13.8	<0.0001	86.96 ± 14.58	82.31 ± 13.78	<0.0001
**Hip circumference (cm)**	92.31 ± 12	94.43 ± 12.36	91.9 ± 11.9	<0.0001	94.39 ± 12.41	91.93 ± 11.88	<0.0001
**WH ratio**	0.9 ± 0.09	0.92 ± 0.09	0.89 ± 0.089	<0.0001	0.92 ± 0.092	0.89 ± 0.089	<0.0001
**Average SBP (mmHg)**	126.63 ± 19.69	134.45 ± 22.12	125 ± 18.75	<0.0001	134.51 ± 22.1	125.08 ± 18.81	<0.0001
**Average DBP(mmHg)**	80.32 ± 11.22	83.89 ± 11.91	79.59 ± 10.94	<0.0001	83.89 ± 11.92	79.62 ± 10.96	<0.0001
**Hemoglobin (g/dl)**	13.21 ± 1.96	12.94 ± 2.2	13.27 ± 1.9	0.001	12.94 ± 2.2	13.27 ± 1.91	0.001
**Fasting blood glucose (mg/dL)**	107.54 ± 46.23	130.65 ± 74.39	102.61 ± 35.95	0.071	130.45 ± 75.1	102.76 ± 35.96	0.109
**Random blood glucose (mg/dL)**	116.59 ± 59.24	131.94 ± 71.8	113.44 ± 55.8	<0.0001	132.62 ± 72.46	113.49 ± 55.82	<0.0001
**Serum creatinine (mg/dL)**	0.82 ± 0.78	1.33 ± 1.73	0.71 ± 0.19	<0.0001	1.34 ± 1.77	0.71 ± 0.19	<0.0001
**eGFR (MDRD) (mL/min/1.73 m**^**2**^**)**	111.31 ± 53	84.27 ± 76.46	116.94 ± 44.65	<0.0001	-	-	-
**eGFR (CKD-EPI) (mL/min/1.73 m**^**2**^**)**	104.9 ± 25.52	-	-	-	82.25 ± 34.95	109.37 ± 20.45	<0.0001
**Urine protein (%)**	13.7	79.5	0	<0.0001	83.4	0	<0.0001
**Hematuria (%)**	19	29.6	16.9	<0.0001	30.4	16.9	<0.0001
**Urine glucose (%)**	8.6	13.5	7.6	<0.0001	14	7.6	<0.0001
**Urine WBCs (%)**	10.4	20.6	10.1	<0.0001	21.5	10	<0.0001

### Prevalence of CKD

**Figure 2:**
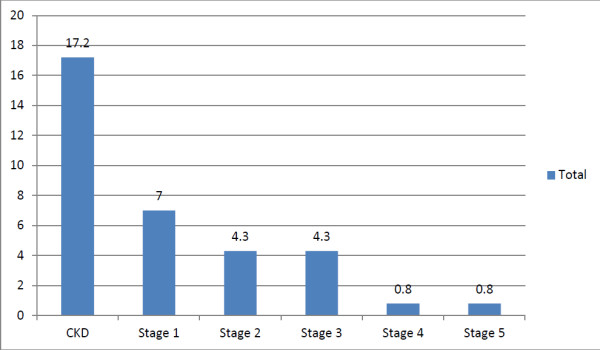
Prevalence of CKD and its stages (using MDRD equation).

Using MDRD equation, the overall prevalence of CKD in the SEEK-India cohort was 17.2% with a mean eGFR of 84.27 ± 76.46 versus 116.94 ± 44.65 mL/min/1.73 m2 in non-CKD group while 79.5% in the CKD group had proteinuria. Prevalence of CKD stages 1, 2, 3, 4 and 5 was 7%, 4.3%, 4.3%, 0.8% and 0.8%, respectively (Figure 2).

**Figure 3:**
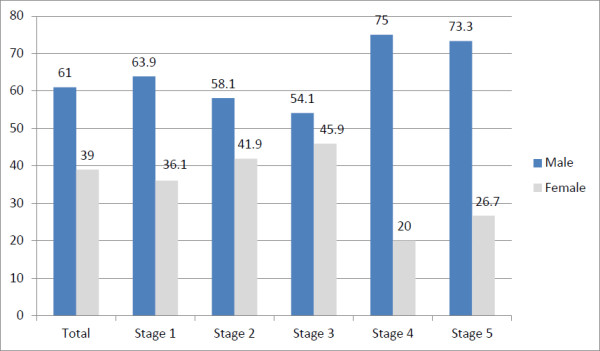
Prevalence of CKD and its stages by gender (using MDRD equation).

CKD was higher in males across all stages of CKD (Figure 3). Those subjects who had low eGFR (<60 ml/min/1.73 m2) comprised 5.9% of the sample (N = 331) while 13.7% had proteinuria (N = 766).

**Figure 4:**
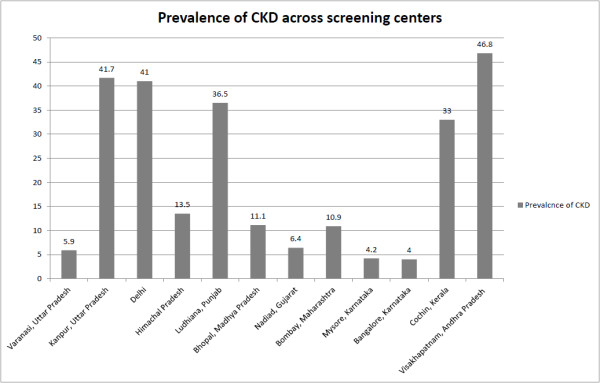
Prevalence of CKD across screening centers.

The prevalence of CKD was center-dependent (Figure 4). The highest prevalence of CKD was observed in Visakhapatnam, Andhra Pradesh (46.8%), Kanpur, Uttar Pradesh (41.7%) and Delhi (41%). The lowest prevalence was observed in Mysore and Bangalore in Karnataka state (4.2% and 4%, respectively).

### CKD Risk factors

**Figure 5:**
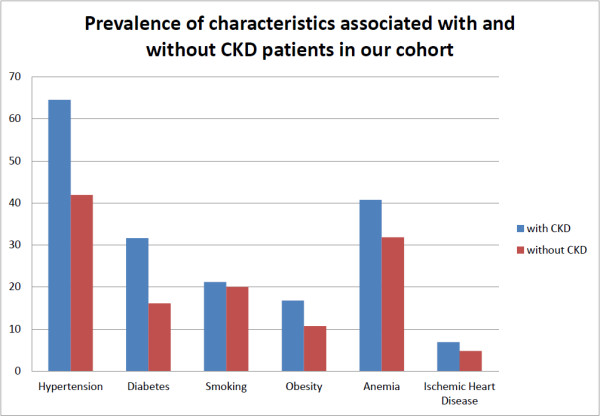
Prevalence of characteristics associated with and without CKD patients in our cohort.

Patients with CKD were older, more likely to be male, more likely to have a high school diploma, more likely to be urban, less likely to have a low income, more likely to be overweight or obese, to have diabetes, hypertension and cardiovascular disease than patients without CKD. The most common risk factors and other characteristics among the subjects diagnosed with CKD were hypertension (64.5%), anemia (40.7%) and diabetes (31.6%) (Figure 5). Anthropometric measures (except height), blood pressure, hemoglobin, random and fasting blood glucose correlated significantly with eGFR in the study cohort. However, age, blood pressure and hemoglobin correlated with eGFR in the CKD subgroup (Table 4).

**Table 4:** Correlations of certain variables with eGFR in the total cohort and in CKD subjects

	**Total cohort**		**CKD subjects**	
	**Spearman’s rho**	***p***	**Spearman’s rho**	***p***
**Age**	−0.454	<0.0001	−0.298	<0.0001
**Height**	0.0	0.993	0.029	0.365
**Weight**	−0.204	<0.0001	0.038	0.243
**BMI**	−0.229	<0.0001	0.015	0.646
**Waist circumference**	−0.286	<0.0001	0.003	0.922
**Hip circumference**	−0.237	<0.0001	0.05	0.146
**Waist/Hip ratio**	−0.196	<0.0001	−0.053	0.12
**SBP**	−0.236	<0.0001	−0.196	<0.0001
**DBP**	−0.159	<0.0001	−0.118	<0.0001
**Hemoglobin**	−0.061	<0.0001	0.263	<0.0001
**Random blood glucose**	−0.156	<0.0001	−0.046	0.177
**Fasting blood glucose**	−0.26	<0.0001	−0.029	0.834

Only 7.9% of the subjects with CKD were aware that they have CKD, while 5.9% of those with proteinuria reported knowing that they had protein in the urine.

### Sensitivity analysis using the CKD-EPI equation

We used the CKD-EPI equation in a sensitivity analysis to test whether it produced similar results to the MDRD-3 equation (Tables 2 and 3). Using the CKD-EPI equation, the prevalence of CKD was 16.4%. CKD Stage 1 was 8%, slightly higher than the one estimated using MDRD-3. However, the prevalence of CKD stages 2 and 3 were 3.2% and 3.3%, respectively and slightly lower than MDRD. The CKD-EPI equation performed similar to MDRD-3 in CKD stages 4 and 5 (0.8% each). A comparison between the CKD and non-CKD subgroups (as defined using the CKD-EPI equation) with respect to the baseline demographics, clinical and laboratory variables, did not yield different results.

## Discussion

The main finding of this study using a convenience cohort design is that the prevalence of CKD in the SEEK-India cohort is 17.2%. The prevalence of CKD stages 1, 2, 3, 4 and 5 was 7%, 4.3%, 4.3%, 0.8% and 0.8%, respectively. Hypertension, anemia and diabetes were the most common risk factors and associated characteristics associated with CKD. Furthermore, old age, hypertension (both SBP and DBP) and low hemoglobin level were correlated with decreased eGFR in the CKD subgroup.

A recent study on 3398 central government employees in India reported ~13-15% prevalence of early stages of CKD (Stages 1, 2 and 3), similar in magnitude to our results, although they used microproteinuria, hematuria and/or leukocyturia as indicators of kidney damage. A study from Apollo Hospital in Chennai [16] reported a prevalence of impaired renal function (eGFR less than 80 mL/min.m2) as 0.86% to 1.39%. This was done through the Rural Program of The Kidney Help Trust of Chennai. The investigators applied a regular screening program of an entire population of 25,000 while treating patients for diabetes and hypertension. Although the Chennai study was a population-based design, it was restricted only residents of a group of villages and hamlets about 50 km away from Chennai [17]. This makes generalization to the entire Indian population difficult. Furthermore, they did not include in their definition of CKD those subjects with proteinuria. In our SEEK-India cohort, 79.5% of those subjects diagnosed with CKD had proteinuria.

Agrawal et al. [18] performed a community-based study to determine the prevalence of CKD in the South Zones of Delhi. They used the multi-stage cluster sampling method in recruiting their subjects. They defined “renal failure” as a serum creatinine >1.8 mg/dL and reported a prevalence of CKD of 0.79%. However, those subjects with positive proteinuria (by dipstick test) constituted 4.4% of their population and were not included in their definition of CKD.

The SEEK-India study is similar in design to the KEEP study conducted by the National Kidney Foundation in the USA [19]. It is a convenience cohort, which invokes both strengths and limitations. In a convenience cohort non-random selection of patients may lead to biases that result in screening of “high risk” individuals thereby inflating the prevalence estimates. On the other hand, our prevalence of 17.2% of CKD is lower to that observed in the NHANES study [20] and is lower than that in the KEEP study [21]. Reports from other Asian countries have shown a high prevalence of CKD. Jafar et al. observed a CKD prevalence of 9% in men and 11% women aged 40 years or over (using NKF criteria) [22].

Reports from other countries participating in the Global SEEK project have been published. The results of SEEK-Thailand study using a cluster randomized design showed 17.5% prevalence of CKD [23]. The SEEK-Saudi Arabia showed a prevalence of 5.7% [24]. Another report from Thailand has shown an increasing prevalence of decreased kidney function using the criterion of GFR <60 ml/min, 1.7% in 1985 to 6.8% in 1997 of the 3499 employees of Electric Generation Authority screened [25]. The prevalence of proteinuria was also increased from 2.64% and 6.10% in the same period. Chen et al. have reported a decreased renal function (GFR < 60 ml/min by the MDRD equation) as 2.53% of 15,540 Chinese adults (35 to 74 years) [26]. The investigators noted that their study has a limitation in that urinary protein was not measured and persons with albuminuria or microalbuminuria were not included in the estimated prevalence of CKD. Therefore, their findings certainly underestimate the prevalence of CKD in the Chinese adult population. In the present study the prevalence of reduced GFR was 5.9% of the screened population. The prevalence of earlier stages of CKD primarily indicated by proteinuria was higher and constituted 13.7%. Results from SEEK-Egypt will be published soon.

Increased prevalence of CKD could be partly explained by the high prevalence of risk factors like diabetes and hypertension in the screened population (18.8% and 431.1%, respectively). The prevalence of diabetes and hypertension in India varied widely in many studies and ranged from 6-20% and 13-58%, respectively [27,28]. Among the CKD group, 64.5% had hypertension and 31.6% had diabetes mellitus. Self reported kidney stones disease was observed in 4.5%.

Despite the high prevalence that we reported in our study, subjects in our cohort had a low awareness of CKD. Only 7.9% of subjects knew that they had CKD compared to 1.9% in SEEK-Thailand and 7.1% in SEEK-Saudi Arabia. This might reflect the lack of healthcare resources available to the population. When performing sensitivity analysis using CKD-EPI equation, the prevalence of CKD in our study was 16.4% (versus 17.2% using MDRD-3 equation). A recently published study aimed at evaluating the applicability of CKD-EPI equation to eGFR in Chinese patients of different stages of CKD [29], compared it with body surface area-standardized GFR (sGFR), which was measured by diethylenetriaminepentaacetic acid renal dynamic imaging method in 142 CKD cases. eGFR was positively correlated with sGFR and the average deviation of eGFR from sGFR was −0.92 ± 16.36 mL/min/1.73 m2 (p = 0.506). They also observed no significant deviation in the CKD from stages 2 to 5. However, in CKD stage 1, the deviation was increased with the value of 13.36 ± 18.44 mL/min/1.73 m [2] (p = 0.023).

In our SEEK-India cohort, the prevalence of CKD was 16.4%. CKD Stage 1 was 8%, slightly higher than the one estimated using MDRD-3 (7%). However, prevalence of CKD stages 2 and 3 were 3.2% and 3.3% respectively, slightly lower than MDRD-3 (4.3% and 4.3% respectively). It also showed similar results to MDRD-3 in CKD stages 4 and 5 (0.8% each). Similar comparisons are supported by other studies in UK [30] where it was shown to be more accurate than the MDRD formula at higher GFR and also reduced the estimated prevalence of CKD stages 3–5 by ~0.5%-0.7%. Further analysis of KEEP study showed similar results [31,32] and the KEEP investigators stated that CKD-EPI will be used to report eGFR in KEEP.

Sixty three percent of our total sample, and almost half of the CKD group (52.6%), earns < $125 a month. Poverty and lack of education often go together. Among the CKD population, 37.6% had less than high school which may have contributed to this lack of awareness. In the NHANES study 11.6% of men and 5.5% of women in CKD stage-3 knew about their disease [20].

Our study had several potential limitations. We used a convenience study design rather than a cluster randomization design and/or household surveys. We mirrored our design on that used by the NKF KEEP cohort in order to bench mark our community based cohort with the results of KEEP. However our sampling strategy may not be ideal for evaluation of true prevalence. Another limitation was the single measurement of serum creatinine and urine albumin. Repeated measures might improve the precision around our reporting of the data. Furthermore, a subsequent measurement after 3 months might have provided additional insights into the chronicity of the disease. Additionally, the prevalence of CKD might have been overestimated by using the Bayer’s multistix 10 which detects urine protein, not albuminuria. Another limitation is that we used the MDRD equation using the race factor for compatible for Americans. However, there are concerns of the application of the definition and staging system for current eGFR estimating equations to the Indian population. Different diet and muscle mass in the Indian as compared to the North American populations may lead to both differences in the normal level for kidney function in the population as well as the relationship between creatinine and GFR as reflected in the estimating equations; where these equations have been predominantly developed and validated.

## Conclusion

In conclusion, in this large, community-based cross-sectional study using a convenience sample of SEEK, we successfully carried out CKD screening using simple tests to estimate e-GFR and protienuria. The prevalence of CKD was observed to be 17.2% with ~6% have CKD stage 3 or worse. CKD risk factors were similar to those reported in earlier studies. Awareness was observed to be low. This data supports the importance of improving the education and early detection of CKD. It should be stressed to all primary care physicians taking care of hypertensive and diabetic patients to screen for early kidney damage. Early intervention may retard the progression of kidney disease. Planning for the preventive health policies and allocation of more resources for the treatment of CKD/ESRD patients are imperative in India.

## Competing interests

The authors declare that they have no competing interest.

## Authors’ contributions

BVM and AKS – conceived and designed the study. AKS – acquired funding, supervised the overall execution of the study. BVM – cleaned the database. YMKF – analyzed and interpreted the data. YMKF, BVM, KKS and AKS – wrote the manuscript. All other co-authors – conducted and supervised the study procedures and the operational execution in the local Indian centers. All authors read and approved the final manuscript.

## Pre-publication history

The pre-publication history for this paper can be accessed here:

http://www.biomedcentral.com/1471-2369/14/114/prepub

## Supplementary Material

Additional file 1 SEEK Project – Screening Questionnaire. Click here for file
