# Supplementary material for: Epidemiology and risk factors of chronic kidney disease in India – results from the SEEK (Screening and Early Evaluation of Kidney Disease) study
Source: BMC Nephrol. 2013 May 28;14:114. doi: 10.1186/1471-2369-14-114 (PMC3848478; doi:10.1186/1471-2369-14-114)
Supplement: Additional file 1 — SEEK Project – Screening Questionnaire. [file 1471-2369-14-114-S1.doc]

**SEEK Project – Screening Questionnaire**

Center ID Camp site

Screening Date Checkin time Data collected by

D M Y

**Personal Information**

**1a**. Participant ID

Camp Number

**1b**. Name

First Name Middle Name / Initials Last Name

2 - Non Tribal

1 - Tribal

**1c**. Category

1 - Male

2 - Female

**1d**. Gender

**1e**. Husband's Name **OR** Father's Name

**1f**. House No. Street Village

Post Taluk

District State PIN

**1g.** Telephone No. Personal PP (tick)

**1h**. Date of Birth **1i**. Age years .

D M Y

1 - Hindu

2 - Christian

3 - Muslim

4 - Sikh

5 - Jain

6 –Other

**1j.** Religion

1 - Unmarried

2 – Married

3 – Divorced

4 – Widow / Widower

**1k.** Marital Status

**1l**. Education

1 - None

2 – Upto 8^th^ Grade

3 – 9^th^ to 12^th^ Grade

4 – Some College

5 – Graduate / Degree

6 – PG / Masters or Professional

**1m** Occupation

1 - Agriculturist

2 - Laborer

3 - Cowherd

4 – Own business / Self employed

5 – Salaried employee

6 - Housewife

7 - Student

8 - Unemployed

9 - Retired

10 - Other

**1n**. Family Income < 2,000Rs./month ,2 to 5000Rs/month; 5 to 10,000rs. /month; >10,000 Rs/month

**1o**. Family consists of members

# Medical Information

**2a.** For women No. of live births No. of miscarriages No. of children alive now

0 - No

1 - Yes

Have you taken birth control pills

1 – High BP

2 - Seizures

3 – Swelling of feet

4 -None

Pregnancy complications

0 - No

1 - Yes

Are you currently menstuating?

**2b.** Have you ever been diagnosed with diabetes or told that your sugar is high?

0 - No

1 - Yes

If yes, how many years ago? Years

-6 – Don’t know

For women, Did you have diabetes before pregnancy?

0 - No

0 - No

-6 – Don’t know

Did you develop diabetes during pregnancy?

1 - Yes

0 - No

1 - Yes

1 - Yes

-6 – Don’t know

Did diabetes persist beyond pregnancy?

0 - None

1 – Oral med

2 – Insulin injection

3 - Both

What medication are you on for diabetes?

Names of the tablets: ____________________________________________________________

0 - No

1 - Yes

Do you have increased frequency of passing urine? If yes, by Day Night

0 - No

1 - Yes

Do you have unusual excessive thirst

0 - No

1 - Yes

0 - No

1 - Yes

Are you on any calorie restricted diet? If yes, for years months

**2c**. Have you ever been told that you have high blood pressure?

If yes, How many years ago was this told / diagnosed? Years

0 - No

1 - Yes

Are you on salt restricted diet? If yes, for years months

0- No

1- yes

What medication are you on for high BP?

Names of the tablets:_______________________________________________________________

0 - No

1 - Yes

-6 – Don’t know

**2d**. Have you ever been told that you have protein or blood in urine?

If yes, How many years ago was this told / diagnosed? Years

0 - No

1 - Yes

0-No

1- yes

Do you have swelling of feet swelling around your eyes / eyelids?

Have you had episodes of burning urine recently? In the past?

0 - No

0 - No

1- yes

1- yes

If yes how long back? How many episodes?

**2e.** Have you had any surgery in the past?

If Yes, Name:________________________________________________________________

0 - No

1 - Yes

**2f.** Have you had any prior medical problems?

If Yes, Name:

# Family History

**3a**. No. of siblings: Brothers Sisters

**3b**. No. of children : born alive No. of children alive today

|  | **Self** | | **Relative( specify the relationship with the patient and No. of relatives affected.)** | |
| --- | --- | --- | --- | --- |
| Hypertension | N | Y | N | Y |
| Diabetes | N | Y | N | Y |
| Heart Attack | N | Y | N | Y |
| Angioplasty | N | Y | N | Y |
| Bypass surgery | N | Y | N | Y |
| Stroke | N | Y | N | Y |
| Burning sensation while urinating | N | Y | N | Y |
| Anemia | N | Y | N | Y |
| Limb amputation | N | Y | N | Y |
| High Cholesterol | N | Y | N | Y |
| Peripheral Vas. Disease | N | Y | N | Y |
| Kidney disease | N | Y | N | Y |
| Kidney stones | N | Y | N | Y |
| Dialysis | N | Y | N | Y |
| Kidney Transplant | N | Y | N | Y |
| Tuberculosis | N | Y | N | Y |

**Note:** Please encircle the relevant ones (N-No, Y-Yes)

# Personal History

0 - Never

1 - Past

2 - Present

**4a**. Do you smoke? cigarettes/beedis/day.Since Yrs

0 - Never

1 - Past

2 - Present

Do you chew tobacco? Since Yrs

1 - Past

2 - Present

**4b**. Do you drink alcohol?

2- Daily

3 - <3days/week

4 - >3days/week

0 - Never

1- Socially

How often Since Years

1 - Vegetarian

2 – Non Vegetarian

**4c.** Food habit:

If non-veg, how often do you have a meal containing meat/egg? Times / month ; Occasionally

How often do you eat outside the house (hotels / fast foods etc)? Times / month ; Occasionally

0 - No

1 - Yes

**4d.** Do you exercise? If yes, How often? Times / week

0 - No

1 - Yes

Does your work involve significant physical activity?( manual labor)

1 – Vigorous-jogging

2 – Moderate – brisk walking

3 – Mild – casual walking

Type of exercise

0 - No

1 - Yes

**4e**. Are you on any allopathic medications other than anti-diabetics ?

If Yes, Name: Purpose:

**4f**. Are you on any homeopathic or ayurvedic medications?

0 - No

1 - Yes

If Yes, Name: Purpose:

**For Physicians**

**5a**. Height (without shoes) cm

**5b**. Weight (without shoes) kg

**5c.** Circumference Waist cm Hip cm

**5d**. Blood pressure: 1^st^ reading Systolic Diastolic mm/Hg

(Sitting) 2^nd^ reading Systolic Diastolic mm/Hg

1- Right

2 - Left

1 – Fore Noon

2 – After Noon

Specify the arm used Time BP measured

1- Occilometric device

2 – Aneroid device

Type of BP apparatus used:

**5e**. Time since last meal Hours Minutes

**5f.** Observations

**For Lab Use**

**6a.** Sample collected on date time

D M Y

**6b**. Analyzed on date

D M Y

**6c**. Hemoglobin gm/dl

**6d**. Serum Glucose mg/dl

**6e**. Serum creatinine mg/dl

**6g**. Urine dipstick:

0 - Negative

1 - Positive

Albumin Semi-quantitation

0 - Negative

1 - Positive

Glucose Semi-quantitation

0 - Negative

1 - Positive

Blood Semi-quantitation

0 - Negative

1 - Positive

Leucocytes Semi-quantitation

**6h.** Spot urine albumin / creatinine ratio

**In case of refusal for enrolment after initial consent:**

1- After questionnaire

2 – After examination

3 – During sample collection

**7a.** Point of refusal

7b. Reason for refusal as expressed by participant __________________________________________

__________________________________________________________________________________

*Checkout time Checked by ___________________ (camp supervisor)*

*Verified by ___________________ (PI / Co-investigator)*
